# Perceptions of pediatric nephrologists regarding timing of dialysis initiation in children in Canada

**DOI:** 10.1186/s40697-016-0123-8

**Published:** 2016-07-01

**Authors:** Jeremy A. Saban, Michael Zappitelli, Susan M. Samuel, Manish M. Sood, R. Todd Alexander, Steven Arora, Robin L. Erickson, Kristine Kroeker, Braden J. Manns, Allison B. Dart

**Affiliations:** McGill University, Montreal, Canada; University of Calgary, Calgary, Canada; University of Ottawa, Ottawa, Canada; University of Alberta, Edmonton, Canada; McMaster University, Hamilton, Canada; University of Saskatchewan, Saskatoon, Canada; University of Manitoba, Winnipeg, Canada; Canadian Kidney Knowledge Translation and Generation Network (CANN-NET), Calgary, Canada; Department of Pediatrics and Child Health, Section of Nephrology, Health Sciences Centre, Children’s Hospital of Winnipeg, FE009 - 840 Sherbrook Street, Winnipeg, MB R3A 1S1 Canada

## Abstract

**Background:**

Significant practice variation exists in Canada with respect to timing of dialysis initiation in children. In the absence of evidence to guide practice, physicians’ perceptions may significantly influence decision-making.

**Objective:**

The objectives of this study are to (1) evaluate Canadian pediatric nephrologists’ perceptions regarding dialysis initiation in children with chronic kidney disease (CKD) and (2) determine the factors guiding practice that may contribute to practice variation across Canada.

**Design:**

This study was a cross-sectional online survey.

**Setting:**

This study was done in academic pediatric nephrology centers in Canada.

**Participants:**

The participants of this study are pediatric nephrologists.

**Measurements and methods:**

An anonymous web-based survey was administered to pediatric nephrologists in Canada to evaluate perspectives and practice patterns regarding timing of dialysis initiation. We also explored the importance of estimated glomerular filtration rate (eGFR) vs. symptoms and the role of patient and provider factors influencing decisions.

**Results:**

Thirty-five nephrologists (59 %) completed the survey. Most respondents care for advanced CKD patients in a multidisciplinary clinic (86 %) and no centers have a formal policy on timing of dialysis initiation. Seventy-five percent of centers follow <20 stage 4–5 CKD patients, and 9 % follow >30 patients. Discussions about dialysis initiation are generally informal (75 %) and the decision to start is made by the nephrologist (37 %) or a team (57 %). Fifty percent agreed GFR was important when deciding when to initiate dialysis, 41 % were neutral, and 9 % disagreed. Variability exists in the threshold that nephrologists considered early (vs*.* late) dialysis initiation: >20 (21 %), >15 (38 %), >12 (26 %), and >10 ml/min/1.73 m^2^ (12 %). Practitioners however *typically* start dialysis in asymptomatic patients at eGFRs of 7–9 (9 %), 10–11 (41 %), 12–14 (38 %), and 15–19 (6 %) ml/min/1.73 m^2^. Patient factors important in the decision to start dialysis for >90 % of nephrologists were fatigue, >10 % weight loss, nausea, increasing missed school, and awaiting a pre-emptive transplant. Age was only a factor for 56 %.

**Limitations:**

This study has a 59 % response rate.

**Conclusions:**

Variability exists in Canada regarding the importance and threshold of eGFR guiding the decision as to when to start dialysis in children, whereas patient symptoms are almost universally important to pediatric nephrologists’ decision-making. Additional studies evaluating outcomes of children starting dialysis earlier vs*.* later are needed to standardize decision-making and care for children with kidney failure.

**Electronic supplementary material:**

The online version of this article (doi:10.1186/s40697-016-0123-8) contains supplementary material, which is available to authorized users.

## Abrégé

**Mise en contexte:**

Des différences significatives existent dans la pratique au Canada quant au moment où des traitements de dialyse devraient être entrepris chez les enfants souffrant d’insuffisance rénale. En absence de données probantes pour guider leur pratique, les différentes approches des néphrologues exerçant auprès de cette population jouent probablement un rôle majeur dans leur prise de décision.

**Objectifs de l’étude:**

Dans un premier temps, l’étude visait à évaluer la perception des néphrologues canadiens en regard de l’amorce de traitements de dialyse chez les enfants atteints d’insuffisance rénale chronique (IRC). Ensuite, on a voulu déterminer les facteurs qui guident la pratique et qui font en sorte que des variations subsistent à cet égard dans la pratique en néphrologie pédiatrique à travers le Canada.

**Cadre et type d’étude:**

Il s’agit d’un sondage transversal mené en ligne auprès des néphrologues pratiquant dans les unités pédiatriques des centres hospitaliers universitaires dans tout le Canada.

**Méthodologie:**

Un sondage accessible par le web, auquel les participants répondaient de façon anonyme, a été distribué aux spécialistes canadiens pratiquant en néphrologie pédiatrique. Ce sondage avait pour objectif d’évaluer les perceptions et les schémas de pratique relativement au moment le plus propice pour amorcer la dialyse. Le sondage explorait aussi l’importance du débit de filtration glomérulaire estimé (DFGe) par rapport aux symptômes ressentis par le patient dans la prise de décision. Finalement, nous avons tenté d’identifier les facteurs pouvant influencer le patient et son médecin traitant au moment de décider d’entreprendre des traitements de dialyse.

**Résultats:**

Tous les pédiatres-néphrologues pratiquant au Canada ont reçu le questionnaire. Toutefois, seulement 35 d’entre eux, soit un peu plus de la moitié (59 %), l’ont complété et renvoyé. La grande majorité des répondants (86 %) exerçaient au sein de cliniques multidisciplinaires, auprès de cas sévères d’IRC. Aucun des centres de soins où ces spécialistes pratiquent ne possédait de politique formelle quant au moment d’entreprendre des traitements de dialyse chez les patients suivis en néphrologie pédiatrique. Les trois quarts des centres de soins cités (75 %) suivaient moins de 20 cas d’IRC de stade 4 ou 5, alors que 9 % en suivaient plus de 30. Les répondants ont indiqué dans une proportion de 75 % que la discussion concernant l’amorce de la dialyse se déroulait de façon informelle. De plus, le sondage révèle que la décision d’amorcer la dialyse est prise par le néphrologue soignant seulement (37 % des cas) ou par une équipe (57 % des cas). La moitié des répondants (50 %) s’accordait pour dire que le DFGe était important dans leur prise de décision d’amorcer des traitements de dialyse chez leurs patients, 41 % avaient une opinion neutre à ce sujet alors que 9 % ne jugeaient pas cet élément important. Les pédiatres-néphrologues répondants sont partagés quant à la valeur seuil de DFGe qu’ils considèrent comme une amorce « hâtive » de dialyse. Pour 21 % des répondants, cette valeur se situe à 20 ml/min/1,73 m^2^; pour 38 % elle se situe plutôt à 15 ml/min/1,73 m^2^; 26 % l’établissent à 12 ml/min/1,73 m^2^; alors que 12 % des répondants jugent l’amorce d’une dialyse dite « hâtive » à un DFGe de 10 ml/min/1,73 m^2^. De façon générale, chez les patients asymptomatiques, la dialyse est amorcée lorsque le DFGe se situe entre 7 et 9 ml/min/1,73 m^2^ (9 % des cas), entre 10 et 11 ml/min/1,73 m^2^ (41 % des cas), entre 12 et 14 ml/min/1,73 m^2^ (38 % des cas) ou entre 15 et 19 ml/min/1,73 m^2^ (6 % des cas). Enfin, parmi les facteurs motivant les patients à entreprendre des traitements de dialyse, tels que rapportés par les répondants dans une proportion de plus de 90 %, on trouve : une sensation de fatigue, une perte de poids de plus de 10 %, des nausées, les absences répétées à l’école et l’attente d’une greffe rénale préventive. L’âge n’a été mentionné comme facteur que dans 56 % des cas.

**Limites de l’étude:**

Le taux de réponse au sondage relativement faible (59 %) limite la portée des résultats.

**Conclusions:**

En néphrologie pédiatrique, alors que les symptômes ressentis par les patients sont considérés de façon universelle dans la décision d’entreprendre une dialyse, les pédiatres-néphrologues canadiens ont des perceptions très différentes quant à la valeur seuil de DFGe qui devrait guider cette décision et à l’importance du rôle que ce paramètre devrait y jouer. Des études supplémentaires comparant les résultats chez les patients qui amorcent une dialyse dite hâtive par rapport à ceux de patients l’amorçant plus tard s’avèrent nécessaires pour établir des pratiques standardisées en matière de soins pour les enfants atteints d’insuffisance rénale.

## What was known before

Dialysis initiation in pediatric chronic kidney disease patients is a relatively rare occurrence. Recent evidence in adults (IDEAL study) shows that there is no benefit to earlier initiation of dialysis, based on eGFR, in terms of survival and quality of life; however, there is little evidence guiding practice in children. In addition, there is significant practice variation amongst pediatric nephrologists across Canada in terms of timing of dialysis initiation based on eGFR.

## What this study adds

This study evaluated perceptions of pediatric nephrologists across Canada regarding timing of dialysis initiation. We have identified important differences in eGFR thresholds considered relevant by pediatric nephrologists and variability in the importance of eGFR in decision-making. This study highlights the higher importance placed on *symptoms* by pediatric clinicians. This study highlights the urgent need for outcome studies in children, to evaluate the health impact of this practice variation.

## Introduction

Dialysis is initiated for an average of 77 incident children each year in Canada [1]. The decision as to *when* to start a child on renal replacement therapy for individual pediatric nephrologists is therefore a relatively rare, yet important and complex decision. Our group has previously shown significant practice variation across the country with respect to timing of dialysis initiation based on the estimated glomerular filtration rate (eGFR) [2, 3]. However, indications for dialysis were not available for this previous study; therefore, an understanding of the factors driving this practice variation remains unknown.

The optimal timing for dialysis initiation in pediatric patients with chronic kidney disease (CKD) remains uncertain. Traditionally, uremic symptoms combined with laboratory data were used to determine when to initiate dialysis in patients with CKD [4]. Throughout the 1970s to the 1990s, starting dialysis at higher eGFRs was thought to be beneficial to patients [5–8]. Thus, a large proportion of CKD patients were beginning dialysis at higher (>10 ml/min/1.73 m^2^) eGFRs across North America throughout the 1990s and early 2000s [9]. However, more recent evidence found that starting dialysis at higher eGFRs could in fact be harmful [10–12]. Moreover, in 2010, the Initiating Dialysis Early and Late (IDEAL) study, a randomized, controlled trial, showed that there was no benefit to early (between 10 and 14 ml/min/1.73 m^2^) initiation of dialysis with respect to survival and quality of life measures in adults [13]. Such a trial has yet to be conducted in children, and there is little data to guide practice with respect to dialysis initiation in children. Furthermore, the applicability of studies such as IDEAL to the pediatric population is unknown.

An understanding of physician perceptions and factors that influence physician decision-making are important in the setting of a paucity of empirical evidence. For example, laboratory parameters including eGFR, physician preferences and knowledge, and individual patient and family characteristics, as well as physician remuneration and healthcare costs, may all affect the decision to initiate dialysis in individual patients. An evaluation of the opinions and practices of pediatric nephrologists concerning when to initiate dialysis will shed light on the factors driving practice variation in Canada and help to guide consensus-based guidelines in children and the design of future intervention trials.

The goals of this study were to (1) evaluate Canadian pediatric nephrologists’ perceptions regarding dialysis initiation in children with CKD and (2) determine the factors guiding practice that may contribute to practice variation across Canada.

## Methods

### Study design, survey development, and administration

This study was a cross-sectional, anonymous, web-based survey. After group discussion (including listed authors) on the goals and target questions of the survey, the survey was designed by AD, reviewed by MZ, and revised by AD. The survey was pilot-tested by two individuals (SS and RE) for general feedback, face validity, clarity, and completion time estimation. Based on this pilot-testing, the final version of the survey was developed. The survey was not evaluated for test-retest reliability. Ethics board approval was granted by the University of Manitoba Bannatyne Campus Research Ethics Board. Consent was presumed based on agreement of participation at the time of survey completion.

Invitation to complete the survey was sent to all 59 pediatric nephrologists in Canada associated with the Canadian Association of Pediatric Nephrologists (CAPN). All CAPN members were contacted via email to participate in an online survey, with two reminder emails sent to all members 2 weeks apart. The survey was administered using a web-based survey program (FluidSurveys™). Two weeks after the second survey reminder invitation, the survey was closed, and the data were exported to Microsoft Excel™ and analyzed.

### Survey content and definitions

The survey was designed to assess demographics, national practice patterns, and perspectives of pediatric dialysis providers regarding the timing of dialysis initiation in children with CKD who lack class indications for immediate dialysis (e.g., encephalopathy, pericardial rub, hyperkalemia, severe metabolic acidosis, fluid overload). The complete survey has been included in Additional file 1 of this publication.

The survey contained two major themes of questions. The first was regarding practitioner demographics and practicing center and program characteristics. The second theme assessed respondents’ opinions regarding dialysis initiation at various thresholds of eGFR under different conditions and situations and in a generic patient with a variety of different characteristics and symptoms. In the survey, questions regarding this theme were posed either with a five-point Likert scale or as ranges of possible eGFRs to choose from.

Early initiation of dialysis was defined as initiating dialysis with an eGFR ≥10.5 ml/min/1.73 m^2^, and late dialysis initiation was defined as an eGFR <10.5 ml/min/1.73 m^2^ based on thresholds utilized in previous observational studies in adults and children [2, 3, 14].

### Statistical analysis

All variables were categorical and reported using descriptive statistics. Given the descriptive nature of this study and the limited sample size, group comparisons were not made.

## Results

Forty of 59 (68 %) Canadian pediatric nephrologists invited to participate responded. Of these 40 respondents, 87.5 % (35) initiated the survey and the average proportion responding to each question was 84 % (33.6 respondents/question).

### Provider demographics

Table 1 outlines the provider characteristics of the respondents. The number of years in practice was evenly distributed between 0 and 5 years to >20 years of practice. With respect to time spent covering the hemodialysis (HD) service, 20 % of the providers cover <7 weeks per year, 54 % cover between 7 and 19 weeks per year, and 26 % cover more than 19 weeks per year. The respondents cover the peritoneal dialysis (PD) service with a very similar time distribution. Over half of the participants (54 %) are reimbursed by an alternate funding plan.

**Table 1:** Provider characteristics of survey respondents

Provider characteristics	Number	Percentage (%)
Years in practice		
0–5	6	17
6–10	10	29
11–15	5	14
16–20	7	20
>20	7	20
Weeks covering hemodialysis		
0–6	7	20
7–12	13	37
13–18	6	17
>19	9	26
Weeks covering peritoneal dialysis^a^		
0–6	7	21
7–12	12	35
13–18	6	18
>19	9	26
Funding model		
Alternate funding plan	19	54
Fee for service	1	3
Mixed model	15	43
Primary way to stay up to date on current literature		
Conferences	6	17
Local rounds presentations/journal clubs	7	20
Regular journal reading	10	29
Journal reading as needed around patient care	12	34

The total number of respondents to the survey was 40. Unless otherwise indicated, the number of respondents to each question was 35

^a^Denotes a question with 34 respondents

### Practice demographics

Table 2 shows the practice characteristics of the respondents. The vast majority (94 %) of the respondents practice in multidisciplinary CKD clinics. Only one respondent reported practicing in a center without a dedicated dialysis program. Eighty percent practice in centers with renal transplantation programs, although 20 and 22 % of HD and transplant programs, respectively, were within adult programs. Over two thirds of the respondents work in centers serving more than 10 prevalent patients with stage 4 or 5 CKD, and only 26 % had >20 patients. The majority of centers had 1–5 HD patients (80 %), and only 14 % had 6–10 active patients. Similarly, 51 % had 1–5 PD patients, and 31 % had 6–10 active PD patients. Only two respondents reported the use of nocturnal hemodialysis at their center. Only 31 % of the respondents stated practicing in centers that have a formal education program for patients and families regarding dialysis and modality selection; none of the respondents reported having a formal policy on dialysis initiation.

**Table 2:** Center characteristics of respondents

Center characteristics	Number	Percentage (%)
Multidisciplinary chronic kidney disease clinics^a^	33	94
Transplant program (pediatric or adult)	28	80
Dialysis program (pediatric or adult)	34	97
Chronic kidney disease population^a^		
>10 patients	23	68
>20 patients	9	26
>30 patients	3	9
Formal patient/family education process regarding dialysis initiation (modality and timing)	11	31
Formal policy on dialysis^a^	0	0

^a^Denotes a question with 34 respondents

### Provider opinions on timing of dialysis initiation

**Fig. 1:**
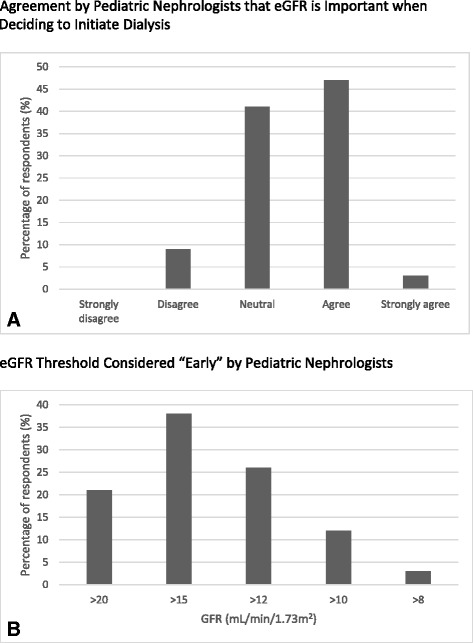
Dialysis provider opinions regarding eGFR in the initiation of dialysis. **a** Vertical bar graph displaying numbers of nephrologists responding to the question “Is GFR important when deciding to start a patient on dialysis?” Responses range from strongly disagree to strongly agree on a five-point scale. The *X* axis represents the possible responses, and the *Y* axis represents the number of respondents. **b** Vertical bar graph displaying numbers of nephrologists responding to the question “What GFR would you consider early initiation of dialysis?” Responses range from >20 to >8 ml/min/1.73 m^2^ on a five-point scale. The *X* axis represents the different GFR response options, and the *Y* axis represents the number of respondents. *GFR* glomerular filtration rate

Fifty percent of respondents agreed or agreed strongly (the two highest options on the five-point Likert scale) that GFR is important in deciding when to initiate dialysis, while 41 % remained neutral (Fig. 1a). Twenty-six percent of the respondents believed that initiating dialysis at a GFR above 12 ml/min/1.73 m^2^ was early, while 38 % and 21 % responded that above 15 ml/min/1.73 m^2^ and above 20 ml/min/1.73 m^2^ were considered early dialysis initiation, respectively. Only 15 % responded that they would consider starting dialysis at an eGFR under 12 ml/min/1.73 m^2^ as an early dialysis initiation (Fig. 1b).

**Fig. 2:**
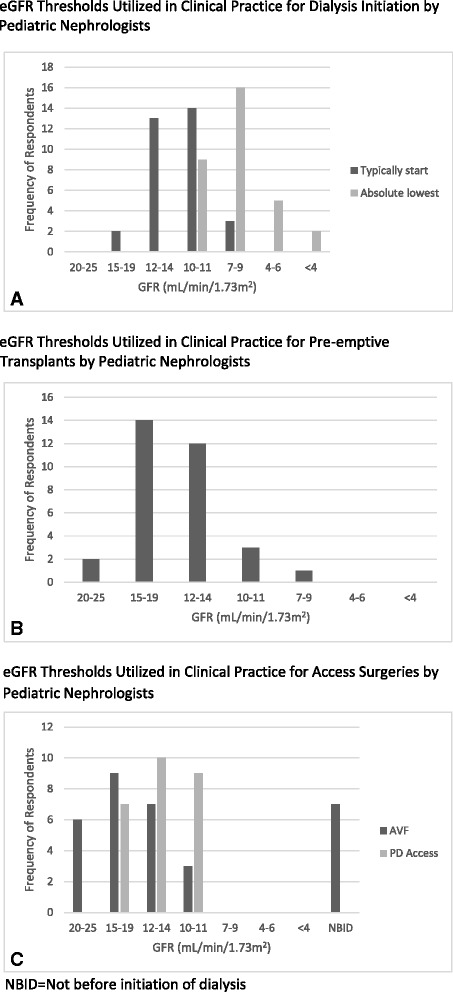
Practice patterns of respondents with regard to dialysis initiation. Vertical bar graphs displaying numbers of nephrologists responding to the questions (**a**) “At what GFR do you typically start dialysis?”, and “What eGFR do you consider to be the absolute lowest you would initiate dialysis in an asymptomatic child?”; **b** “At what GFR would you consider a pre-emptive transplant in an asymptomatic child?”; and **c** “When would you insert an arterio-venous fistula in an asymptomatic child?” and “When would you insert a peritoneal dialysis access in an asymptomatic child?” Responses are represented on the *X* axis and range from 20 to 25 ml/min/1.73 m^2^ to <4 m/min/1.73 m^2^ with seven range options. The *Y* axis represents the number of respondents. *NBID* not before initiation of dialysis, *GFR* glomerular filtration rate

Figure 2 shows the typical practice patterns of the respondents with regard to dialysis initiation. Figure 2a shows that 91 % of dialysis providers *typically* initiate dialysis in their patients at an eGFR of 10 ml/min/1.73 m^2^ or above. However, when asked what eGFR they considered the absolute lowest they would initiate dialysis in an asymptomatic child, 72 % gave responses below 10 ml/min/1.73 m^2^. Furthermore, 97 % of nephrologists responded that they would consider a pre-emptive transplant in an asymptomatic child with an eGFR of 10 ml/min/1.73 m^2^ or greater, and 50 % would do so at an eGFR >14 ml/min/1.73 m^2^ (Fig. 2b). The respondents would consider an arterio-venous fistula (AVF) as early as an eGFR of 20–25 ml/min/1.73 m2; however, the earliest a PD catheter would be inserted was reported at an eGFR <20 ml/min/1.73 m^2^ and most commonly at an eGFR between 10 and 14 ml/min/1.73 m2 (Fig. 2c).

**Fig. 3:**
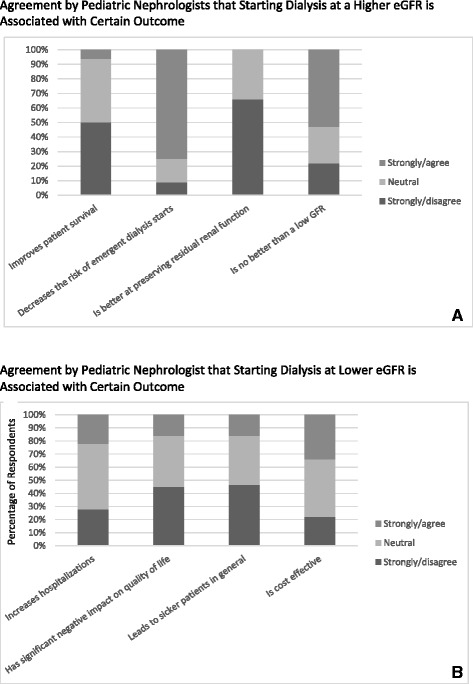
Summary of provider opinions regarding dialysis initiation at high and low GFRs. Stacked percentage graph displaying percentage of total respondents opinions regarding (**a**) starting dialysis at high GFRs and (**b**) beginning dialysis at low GFRs. Responses range from strongly disagree to strongly agree on a five-point scale. Strongly agree and agree, as well as strongly disagree and disagree, responses were grouped together for ease of interpretation. The *X* axis shows the different prompts evaluated by respondents, while the *Y* axis shows the percentage of the total respondent opinions regarding each prompt

Figure 3a summarizes providers opinions regarding starting dialysis at a high eGFR (early, defined as >10.5 ml/min/1.73 m^2^). When asked if starting dialysis at a high eGFR improves patient survival, 50 % disagreed or disagreed strongly (the two lowest points on the five-point Likert scale). Only 9 % of the respondents disagreed or disagreed strongly that it decreases the risk of emergent dialysis. Two thirds of the respondents disagreed or disagreed strongly that initiation of dialysis at high eGFR better preserves renal function. Only 22 % disagreed or disagreed strongly that in terms of clinical outcomes, high eGFR dialysis initiation is no better than low eGFR dialysis initiation.

Figure 3b assesses providers opinions regarding starting dialysis at a lower eGFR. When asked if starting dialysis at a low eGFR increases hospitalizations, 22 % agreed or strongly agreed. Furthermore, only 16 % of the respondents agreed or agreed strongly that it has a negative impact on the quality of life. Similarly, 16 % of the respondents agreed or agreed strongly that late initiation by eGFR criteria leads to sicker patients. Finally, 34 % agreed or agreed strongly that starting dialysis at a low eGFR is cost-effective.

When asked if age was an important factor in their decision to start dialysis, the respondents were split evenly between yes and no. For those who said yes, most respondents said that they would initiate dialysis *earlier* in a younger child. Seventy-four percent of the respondents considered less than 1 year of age an important threshold in decision-making. Furthermore, 55.5 % considered this threshold to be <3 months of age. Fifteen percent would also consider *no*t starting dialysis on a very young child, whereas 61.8 % would only consider not starting dialysis if there were comorbidities affecting the quality of life or life expectancy, prematurity, a size <2.5 kg, or a family choice not to start.

We additionally assessed provider’s opinions regarding available adult literature. In response to whether providers would change clinical practice based on adult literature, a great majority responded yes, as long as the results are robust and valid and could apply to children (83 %). However, when directly asked whether the IDEAL trial altered their practice, only 25 % of the respondents said yes, 62.5 % said no, and 12.5 % were not familiar with the trial.

### Provider opinions on patient characteristics and symptoms important to dialysis initiation

Table 3 displays the patient characteristics and symptoms that were reported as most important to respondents for decision-making in dialysis initiation in outpatients with progressive CKD. Fatigue (100 %), missed school days (97 %), weight loss of greater than 10 % (94 %), nausea (94 %), and pruritis (85 %) were nearly unanimously agreed upon as important or very important. Other less specific factors such as young patient age (56 %), inability to keep up in sports (53 %), and patient preference (53 %) were agreed upon by approximately half of the respondents. Factors reported as being less important to the respondents included selection of peritoneal dialysis modality (32 %), etiology of ESRD (21 %), and poor patient adherence (21 %).

**Table 3:** Importance of patient characteristics and uremic symptoms for pediatric nephrologists with respect to timing of dialysis

Characteristics	Number	Percentage rating of important/very important (%)
Fatigue	34/34	100
Increased missed school days	33/34	97
Weight loss >10 %	32/33	94
Nausea	32/34	94
Pruritis	29/34	85
Weight loss >5 %	25/33	74
Suboptimal height velocity	24/34	71
Young patient age	19/34	56
Inability to keep up in sports	18/34	53
Patient/family preference	18/34	53
Peritoneal dialysis modality	11/34	32
Etiology of end stage renal disease	7/34	21
Poor patient adherence	7/34	21

## Discussion

This is the first report of a national survey of pediatric nephrologists to evaluate opinions and factors driving practice concerning dialysis initiation in children. This study has importantly identified that only 50 % of pediatric nephrologists in Canada consider GFR important when deciding the timing of dialysis initiation in their patients. There is also significant variability in the threshold that nephrologists consider early (vs*.* late) initiation with almost 60 % choosing a threshold above 15 ml/min/1.73 m^2^ and only 12 % choosing >10 ml/min/1.73 m^2^. In contrast, the threshold at which practitioners *typically* start asymptomatic children on dialysis is between 10 and 14 ml/min/1.73 m^2^ in most cases, a threshold now considered “high or early” in the adult literature. Important clinical factors have emerged as more relevant triggers for dialysis initiation by pediatric care providers including fatigue, weight loss, nausea, and increasing school absenteeism. Awaiting a pre-emptive transplant is also an important factor that can delay the decision to start dialysis. These findings shed important light on the reasons for variability in practice across Canada previously reported by our group [2, 3].

The previous pediatric literature on the topic of timing of dialysis initiation in children includes retrospective analyses of large databases in North America [15, 16] and Europe [17, 18], which have reported that 50 % of children are started on dialysis with an eGFR >10 ml/min/1.73 m^2^ and about 20 % started with an eGFR >15 ml/min/1.73 m^2^. Our group also reported, utilizing the Canadian Organ Replacement Register (CORR) data, that 30 % of children in Canada are started on dialysis with an eGFR ≥10.5 ml/min/1.73 m^2^. This study showed considerable practice variation across the country, with a range of 12–70 % of children started with an eGFR >10.5 ml/min/1.73 m^2^ by treatment facility [3]. The limitation of these previous studies is a lack of information that may have influenced clinical decision-making, such as the clinical indication for dialysis and the presence of uremic symptoms at the time of dialysis initiation.

This survey provides insight into the likely explanations for the previously identified variation in practice based on eGFR, as practitioners are in fact not utilizing the same target eGFR thresholds nor do they universally agree that eGFR should be an important tool in the decision-making process to begin with. This suggests that future studies on timing of dialysis initiation in children based on eGFR may be challenging to perform. Although the IDEAL trial in adults has rigorously shown a lack of clinical benefit to starting dialysis at a higher eGFR (eGFR 10–14 vs. 5–7 ml/min/1.73 m^2^) [13], the applicability of these findings to children is at this time unknown. While most respondents did report changing their pediatric clinic practice based on adult literature, the lack of translation of the adult trial evidence on timing of dialysis initiation specifically into pediatric practice is explicit. This was demonstrated by two thirds of the respondents stating the IDEAL trial did not alter their practice.

A range of opinions were uncovered in this survey, especially in regard to factors influencing later initiation of dialysis. Respondent answers were mixed as to whether late dialysis initiation increases hospitalizations, has negative impacts on the quality of life, leads to sicker patients, or is cost-effective. This reflects the lack of evidence in this area in pediatrics and a need for outcome studies to address these significant knowledge gaps. The only outcome-based study that has been published to date revealed a 21 % decreased risk of hospitalizations for hypertension and pulmonary edema in children with higher baseline eGFRs at dialysis initiation [16]. However, this study was observational, and differences between groups may reflect the impact of unmeasured confounders. Whether early dialysis initiation is harmful or not is not ascertainable from the available evidence, and clearly, more research is required in order for pediatric nephrologists to make informed decisions.

The importance of patient factors such as symptoms of uremia in decision-making was universally supported in the survey. When assessing what factors were important or very important to dialysis providers in determining when to start dialysis, three tiers of responses emerged. Most respondents agreed that uremic symptoms such as fatigue, nausea, and pruritis were important in decision-making. In the same tier were measurable functional indicators such as increased missed school days, weight loss >10 %, and suboptimal height growth velocity. These factors are all similar in their objective and measurable nature. On the other hand, the more subjective, less measurable factors fell to a lower tier of importance to providers. This indicates a clear preference of physicians for more objective factors in determining when to initiate dialysis.

This survey also evaluated important facility and practice characteristics that could influence the feasibility of future intervention trials in children. Firstly, local pediatric CKD and dialysis populations are quite small. Therefore, the ability to perform single-center studies does not exist, and all research efforts will require national and international collaborations to obtain sufficient sample sizes.

Despite small program sizes, most centers do function with a multidisciplinary CKD clinic and have the ability to perform dialysis and transplants within their local program; therefore, resource issues should not be significant factors influencing dialysis starts. However, some areas for improvement identified by the survey include only 31 % of the respondents are using a patient and family educational process, and there is a universal lack of formal policies on timing of dialysis initiation. The reason for this lack of formalized decision-making process is likely multifactorial, owing to a lack of evidence from the literature, a lack of pediatric studies regarding dialysis initiation, or perhaps due to a deficiency in knowledge translation from the adult literature.

Our study was not without limitations. We had approximately a two-third response rate to our survey. However, the distribution of the respondents came from all parts of the country and is therefore likely to be representative of the spectrum of pediatric nephrologists in Canada. Though 40 physicians responded to the survey, the most responses received for any one question was 35. Excluding these five individuals still leaves a response rate of 59 %. Our survey was limited by a lack of power to statistically assess the provider or facility characteristics that may be influencing eGFR thresholds. Dialysis initiation is clearly a decision made through the input of several important groups including the physicians, nurses, patients, parents, and the facilities themselves. Outside of the scope of this project but an important limitation was the lack of responses by parents, patients, and nurses involved in the decision to initiate dialysis. More research is necessary to understand the roles of each of these groups in the decision-making process for dialysis initiation. Finally, our survey mostly pertains to children with slowly progressive CKD, as opposed to those who present with acutely falling GFRs and classical indications for dialysis. As a result, many respondents may have envisioned older children when formulating their answers in this survey. Nonetheless, only 50 % thought that age was an important factor in their decision-making and would start dialysis at an earlier threshold in children less than 1 year of age. Therefore, the findings likely apply to all children over 1 year of age.

## Conclusions

Variability exists in Canada regarding the importance and threshold of eGFR guiding the decision as to when to start dialysis in children, whereas patient symptoms are almost universally important to pediatric nephrologists’ decision-making. Importantly, numerous knowledge gaps exist in our understanding of what factors *should* determine when to initiate renal replacement therapy in children with advanced CKD in order to optimize patient outcomes. Additional studies evaluating outcomes of children starting dialysis earlier vs*.* later are needed to standardize decision-making and care for children with kidney failure.

## Additional file

Additional file 1: An assessment of dialysis provider’s attitudes towards timing of dialysis initiation in children. Survey questions completed by participants. (DOCX 207 kb)
