# Supplementary material for: Perceptions of pediatric nephrologists regarding timing of dialysis initiation in children in Canada
Source: Can J Kidney Health Dis. 2016 Jul 1;3:31. doi: 10.1186/s40697-016-0123-8 (PMC4929756; doi:10.1186/s40697-016-0123-8)
Supplement: Additional file 1: — An assessment of dialysis provider’s attitudes towards timing of dialysis initiation in children. Survey questions completed by participants. (DOCX 207 kb) [file 40697_2016_123_MOESM1_ESM.docx]

An assessment of dialysis provider’s attitudes towards timing of dialysis initiation in children

# Purpose:

# 1. To assess national practice patterns of pediatric dialysis providers regarding the timing of dialysis initiation

# 2. To assess current practice, as well as the perspective of pediatric dialysis providers regarding the timing of dialysis initiation.

# Aims:

# 1. To determine center and dialysis provider factors associated with timing of dialysis initiation

# 2. To assess dialysis' providers attitudes regarding the evidence on the advantages and disadvantages of early versus late dialysis initiation

# This survey is approved by the University of Manitoba Research Ethics Board. Data will only be published in aggregate form.

# Part 1: Provider's characteristics.

# In what city do you practice?

# How many years have you been in practice?

|  | 0-5 years |
| --- | --- |
|  | 6-10 years |
|  | 11-15 years |
|  | 16-20 years |
|  | >20 years |

# How many weeks per year do you provide care to patients receiving hemodialysis?

|  | 0 weeks |
| --- | --- |
|  | 1-6 weeks |
|  | 7-12 weeks |
|  | 13-18 weeks |
|  | > 19 weeks |

# How many weeks per year do you provide care to patients receiving peritoneal dialysis?

|  | 0 weeks |
| --- | --- |
|  | 1-6 weeks |
|  | 7-12 weeks |
|  | 13-18 weeks |
|  | > 19 weeks |

# Do you use an electronic medical record (EMR) to provide care to your CKD patients?

|  | Yes |
| --- | --- |
|  | No |

# Do you use a PDA to guide care for your CKD patients?

|  | Yes |
| --- | --- |
|  | No |

# What is your funding model?

Alternate funding plan

Fee for service

Mixed model

# What is the primary way that you stay up to date on the current literature?

|  | Conferences |
| --- | --- |
|  | Local rounds presentations/journal club |
|  | Regular journal reading |
|  | Journal reading as needed around patient care |

# Do you change your pediatric clinical practice based on adult literature?

|  | Yes, if the adult study is robust and valid. |
| --- | --- |
|  | Yes, if results are robust and valid and could apply to children. |
|  | No, never. I wait for pediatric studies to be published. |

# Part 2: Practice characteristics.

# At your centre, in what setting are stage 4-5 CKD patients managed?

|  | In a multidisciplinary clinic with one consistent physician, one consistent nurse. |
| --- | --- |
|  | In a multidisciplinary clinic with one consistent physician, different nurse. |
|  | In a multidisciplinary clinic with a different physician (group practice), one consistent nurse. |
|  | In a multidisciplinary clinic with an different physician (group practice), different nurse. |
|  | In general nephrology clinics (not multidisciplinary). |
|  | Other ______________________ |

# How does your centre educate patients and families regarding dialysis +/- modaliy selction? (check all that apply)

|  | Informal discussion in clinical setting primarily by nephrologist. |
| --- | --- |
|  | Informal discussion in clinical setting primarily by nurse clinician/educator. |
|  | Formal (standard) presentation by physician. |
|  | Formal (standard) presentation by nurse clinician/educator. |
|  | Patient/family support groups. |
|  | Other ______________________ |

# Does your centre have a transplant program?

|  | Yes, pediatric specific |
| --- | --- |
|  | Yes, within adult program |
|  | No |

# Does your centre have a hemodialysis program/unit?

|  | Yes, pediatric specific. |
| --- | --- |
|  | Yes, within adult program |
|  | No |

# At your centre how is the decision made as to WHEN to start a new patient on dialysis? (Assuming discussion with patient and family occurs at all levels).

|  | By nephrologist in clinical setting. |
| --- | --- |
|  | Informal discussion between 2+ nephrologists. |
|  | In a team meeting format including 2+ nephrologists, allied health practitioners. |
|  | In a team meeting format with all relevant section members. |

# Are physicians remunerated more for hemodialysis than for peritoneal dialysis in your region of practice?

|  | Yes |
| --- | --- |
|  | No |

# Does your centre have a surgeon(s) or alternate (interventional radiology) that can insert:

|  | Yes | No |
| --- | --- | --- |
| PD catheters |  |  |
| Central venous catherters |  |  |
| A-V fistulas |  |  |

# Is there a higher remuneration fee for caring for patients with severe CKD?

|  | Yes |
| --- | --- |
|  | No |
|  | Don't know |

# Approximately how many patients in each category are cared for in your centre:

| CKD stage 4-5? | \|  \| 0  1-5 \| \| --- \| --- \| \|  \| 6-10 \| \|  \| 11-15 \| \|  \| 16-20 \| \|  \| 21-25 \| \|  \| 26-30  >30 \| |
| --- | --- | --- | --- | --- | --- | --- | --- | --- | --- | --- | --- | --- | --- |
| Peritoneal dialysis? | \|  \| 0  1-5 \| \| --- \| --- \| \|  \| 6-10 \| \|  \| 11-15 \| \|  \| 16-20 \| \|  \| 21-25 \| \|  \| 26-30  >30 \| |
| Conventional HD? | \|  \| 0  1-5 \| \| --- \| --- \| \|  \| 6-10 \| \|  \| 11-15 \| \|  \| 16-20 \| \|  \| 21-25 \| \|  \| 26-30  >30 \| |
| In-centre nocturnal HD? | \|  \| 0  1-5 \| \| --- \| --- \| \|  \| 6-10 \| \|  \| 11-15 \| \|  \| 16-20 \| \|  \| 21-25 \| \|  \| 26-30  >30 \| |
| At home nocturnal HD? | \|  \| 0  1-5 \| \| --- \| --- \| \|  \| 6-10 \| \|  \| 11-15 \| \|  \| 16-20 \| \|  \| 21-25 \| \|  \| 26-30  >30 \| |
| In-centre short daily HD? | \|  \| 0  1-5 \| \| --- \| --- \| \|  \| 6-10 \| \|  \| 11-15 \| \|  \| 16-20 \| \|  \| 21-25 \| \|  \| 26-30  >30 \| |
| At home short daily HD? | \|  \| 0  1-5 \| \| --- \| --- \| \|  \| 6-10 \| \|  \| 11-15 \| \|  \| 16-20 \| \|  \| 21-25 \| \|  \| 26-30 \| |
| Long conventional HD (>5 hours three times a week)? | \|  \| 0  1-5 \| \| --- \| --- \| \|  \| 6-10 \| \|  \| 11-15 \| \|  \| 16-20 \| \|  \| 21-25 \| \|  \| 26-30  >30 \| |
| a combination of above modalities? | \|  \| 0  1-5 \| \| --- \| --- \| \|  \| 6-10 \| \|  \| 11-15 \| \|  \| 16-20 \| \|  \| 21-25 \| \|  \| 26-30  >30 \| |

# Approximately how many *half* days per week is your hemodialysis unit open?

If you are unsure, please write unsure in the box below.

# Approximately how many hemodialysis stations are in your unit?

If you are unsure, please write unsure in the box below.

# Part 3: Timing of dialysis

# Does your centre have a formal program-wide policy on when to start patients on dialysis?

|  | Yes |
| --- | --- |
|  | No  Don’t know |

# If yes to the question above, is this policy based on:

|  | eGFR alone? |
| --- | --- |
|  | eGFR and symptoms? |
|  | Symptoms only? |

# How do you predominantly assess renal function in late stages of CKD to guide decisions regarding dialysis initiation?

|  | eGFR (Schwartz)  Cystatin C |
| --- | --- |
|  | 24 hr urine for CrCl |
|  | Nuclear GFR |
|  | Iohexol GFR |
|  | Combination of estimated and measured GFR |

# How often do you perform a nuclear GFR or an iohexol GFR on a patient with stage 4 CKD?

|  | Never |
| --- | --- |
|  | Once per year routine |
|  | Every 6 months routine |
|  | Every 2 years routine |
|  | When felt to be clinically indiacated only |

# GFR is very important when deciding when to start a patient on dialysis.

|  | Strongly disagree | Disagree | Neutral | Agree | Strongly Agree |
| --- | --- | --- | --- | --- | --- |
|  |  |  |  |  |  |

# If you had to choose, what eGFR threshold would you consider an early initiation of dialysis?

|  | >20ml/min/1.73m2 |
| --- | --- |
|  | >15ml/min/1.73m2 |
|  | >12ml/min/1.73m2 |
|  | >10ml/min/1.73m2 |
|  | >8ml/min/1.73m2 |

# At what eGFR in an asymptomatic child would you insert a AVF hemodialysis access?

|  | 20-25 |
| --- | --- |
|  | 15-19 |
|  | 12-14 |
|  | 10-11 |
|  | 7-9 |
|  | 4-6 |
|  | <4 |

# At what eGFR in an asymptomatic child would you insert a peritoneal dialysis access?

|  | 20-25 |
| --- | --- |
|  | 15-19 |
|  | 12-14 |
|  | 10-11 |
|  | 7-9 |
|  | 4-6 |
|  | <4 |

# At what eGFR in an asymptomatic child would you consider doing a pre-emptive transplant?

|  | 20-25 |
| --- | --- |
|  | 15-19 |
|  | 12-14 |
|  | 10-11 |
|  | 7-9 |
|  | 4-6 |
|  | <4 |

# At what eGFR do you typically start dialysis?

|  | 20-25 |
| --- | --- |
|  | 15-19 |
|  | 12-14 |
|  | 10-11 |
|  | 7-9 |
|  | 4-6 |
|  | <4 |

# Is there an absolute lowest eGFR in an asymptomatic child on whom you would start dialysis?

|  | 20-25 |
| --- | --- |
|  | 15-19 |
|  | 12-14 |
|  | 10-11 |
|  | 7-9 |
|  | 4-6 |
|  | <4 |

# In the absence of classical indications for dialysis (hyperkalemia, symptomatic fluid overload, severe metabolic acidosis, uremic pericarditis, encephalitis), rate the importance of the following clinical features with respect to their impact on your decision to start dialysis “early”

|  | Very unimportant | Unimportant | Neutral | Important | Very important |
| --- | --- | --- | --- | --- | --- |
| Young patient age |  |  |  |  |  |
| Increasing fatigue |  |  |  |  |  |
| Suboptimal height velocity |  |  |  |  |  |
| Weight loss (>5%) |  |  |  |  |  |
| Weight loss (>10%) |  |  |  |  |  |
| Nausea |  |  |  |  |  |
| Pruritus |  |  |  |  |  |
| Increased missed school days due to symptoms |  |  |  |  |  |
| Inability to "keep up" in sports |  |  |  |  |  |
| Poor patient adherence |  |  |  |  |  |
| Patient/family preference |  |  |  |  |  |
| Severe nephrotic syndrome |  |  |  |  |  |
| Lack of Polyuria  Peritoneal dialysis modality  Etiology of ESRD |  |  |  |  |  |

# Rate your agreement with the following potential rationales for starting dialysis at a "late" eGFR .

|  | Strongly disagree | Disagree | Neutral | Agree | Strongly agree |
| --- | --- | --- | --- | --- | --- |
| Awaiting pre-emptive transplant |  |  |  |  |  |
| Concerned about patient's ability to tolerate dialysis treatment safely secondary to behavioral issues/development delay |  |  |  |  |  |
| Patient/family preference |  |  |  |  |  |
| Distance of patient from health care facility  Peritoneal dialysis modality |  |  |  |  |  |

# What is the most important uremic clinical symptom that determines when it is time to start dialysis in a child? (open ended)

# Does age alter your decision to start dialysis?

Yes

No

**If yes to question above, do you start younger patients earlier?**

Yes

No

**Would you consider not starting dialysis at all based on very young age?**

Yes

No

Depends on ___________

**At what threshold do you take age into consideration?**

<1mo

<3mo

<6mo

<1 year

<2 years

<5 years

<10 years

<14 years

<16 years

<18 years

# Part 4: Provider’s attitudes

# For outpatients with progressive CKD, do you agree or disagree with the following statements?

|  | Strongly disagree | Disagree | Neutral | Agree | Strongly agree |
| --- | --- | --- | --- | --- | --- |
| Starting dialysis with a high GFR improves patient survival. |  |  |  |  |  |
| Starting dialysis with a low GFR increases hospitalizations. |  |  |  |  |  |
| Starting dialysis with a high GFR decreases the risk of emergent dialysis starts. |  |  |  |  |  |
| Starting dialysis with a low GFR has significant negative impact on QOL. |  |  |  |  |  |
| Starting dialysis with a low GFR leads to sicker patients in general. |  |  |  |  |  |
| Starting dialysis with a low GFR is cost effective. |  |  |  |  |  |
| Starting dialysis with a high GFR is better at preserving residual renal function |  |  |  |  |  |
| In terms of clinical outcomes, initiating dialysis at a high GFR is no better than a low GFR. |  |  |  |  |  |

# Did the results from the IDEAL trial (no difference in mortality between those randomized to a high and low eGFR for initiation of dialysis) alter your practice?

|  | Yes |
| --- | --- |
|  | No |
|  | I'm not familiar with that trial. |
